# Modelling of the impact of universal added sugar reduction through food reformulation

**DOI:** 10.1038/s41598-017-17417-8

**Published:** 2017-12-12

**Authors:** Chris Ho Ching Yeung, Paayal Gohil, Anna M. Rangan, Victoria M. Flood, Jayashree Arcot, Timothy P. Gill, Jimmy Chun Yu Louie

**Affiliations:** 1 School of Biological Sciences, Faculty of Science, The University of Hong Kong, Pokfulam, Hong Kong; 2 Charles Perkins Centre and School of Life and Environmental Sciences, Faculty of Science, The University of Sydney, Sydney, NSW Australia; 3 Faculty of Health Sciences, The University of Sydney, Sydney, NSW Australia; 4 School of Chemical Engineering, Faculty of Engineering, The University of New South Wales, Kensington, NSW Australia; 5 Boden Institute of Obesity, Nutrition, Exercise and Eating Disorders, The University of Sydney, Sydney, NSW Australia

## Abstract

Food reformulation has been suggested to be one of the strategies to reduce population added sugar (AS) intake. This study aims to investigate the untested assumption that a reduction in AS through reformulation will result in a reduction in population intakes of AS and energy. Plausible dietary data from 4,140 respondents of an Australian national nutrition survey were used. Dietary modelling was performed at AS reductions of 10%, 15%, and 25% using four strategies: simple removal of AS or replacement with non-nutritive sweeteners (NNS), and replacement of AS with NNS and either: polyols, 50% fibres or 50% maltodextrin. Paired t-tests were conducted to compare the intake of energy, fat, and AS pre- and post-reformulation. The chosen reformulation strategies resulted in a projected reduction in AS and energy, with the greatest reduction found in 25% reformulation which was the highest level modelled. The overall projected mean (SD) reduction in energy and AS after 25% reformulation was 114 (92) kJ/day and 11.73 (7.52) g/day, *p* < 0.001. To conclude, product reformulation may be a potentially useful strategy for reducing AS intake. Although the magnitude of projected reduction was small at the individual level, the impact may be meaningful at a population level.

## Introduction

Added sugars are commonly defined as those that are added to food during processing, preparation, or at the table. As added sugars contribute energy (kilojoules) to the diet but have little nutritional benefit, high intakes are thought to be associated with diluted nutrient density^1^, increased energy content of diet^1^, dental caries^2^, and other adverse health outcomes such as excess weight gain and reduced bone strength^3^.

In light of this, the World Health Organisation (WHO) recommends a reduction in ‘free’ sugars (added sugars plus sugars from fruit juices) in the diet to reduce the prevalence of diet-related chronic disease. The current WHO recommendations state that ‘free’ sugars should be less than 10% of total energy intake, and less than 5% for additional health benefits based on evidence regarding the relationship between free sugar and body weight/dental caries^2^. Nonetheless, our group has recently reported that more than 70% of Australian children and adolescents exceeded the 10% cut-off, with the majority of their daily added sugar intake coming from high sugar discretionary foods such as sugar-sweetened beverages, cakes and biscuits^4^. Therefore, it is clear that the diet of Australian children and adolescents could be improved to lower their added sugars intake.

It has been argued that the amount of added sugars in packaged foods is high. Food reformulation has been suggested to be a potentially useful option to reduce the population added sugars intake, as it allows minor yet positive changes to be made to diets without consumers making major changes to their dietary patterns^5^. This was largely based on the success with salt reformulation. For example, He, Brinsden and MacGregor^6^ reported that the UK salt reduction program has resulted in reductions in the salt content of processed foods, and a 15% reduction in 24-h urinary sodium (from 9.5 g to 8.1 g/d) in a 7 year period. A recent study in UK also suggested that a gradual reduction of 40% free sugar in sugar sweetened beverages (SSBs) through a 5 year period could reduce on average 38.4 kcal/day (161 kJ/day) energy intake^7^.

However, reduction in added sugars content in processed food could be more challenging as sugars play a variety of roles in processed foods other than just providing sweetness. These include, but are not limited to, provision of colour, bulk, and texture; enhancing flavour; and acting as a preservative^8^. Some of these functions are not easy to replace with an alternative ingredient^8^. In addition, when sugars are removed from the food product without being replaced with another ingredient, the remaining ingredients will ‘concentrate’ on a per 100 g basis. As consumers will likely eat a similar weight of the reformulated product compared with the original product, such ‘concentration’ effects of ingredients on nutrient intakes should be examined.

Given the uncertainties regarding the potential positive and negative impacts of adjusting the added sugars content of foods and drinks, the current study aims to investigate the theoretical effects of reformulating processed foods (at three different levels of added sugar reduction: 10%, 15% and 25%) on intakes of energy, added sugar, total fat and saturated fat of Australian children and adolescents. Four reformulation strategies were chosen on the basis of their potential ability to replace the functional roles of added sugar in processed food as suggested in previous studies^9^. These models include: simple removal with no replacement/replacement with non-nutritive sweeteners (NNS) only; replacement with polyols & NNS; 50% fiber & NNS; and 50% maltodextrin & NNS.

## Results

### Subject characteristics

Table 1 summarized the subject characteristics. The mean (SD) BMI of the study population was 18.5 (3.7) kg/m^2^. The majority of respondents lived in an urban area. The mean intake of added sugars and energy ranged from 33.0 (20.5) g and 5 913 (1 177) kJ for 2–3 years old girls to 85.6 (44.9) g and 11 574 (2 644) kJ for 14–16 years old boys.

**Table 1:** Subject characteristics and daily dietary intake of energy and selected macronutrients, stratified by age groups and sex.

	Age groups									
	2–3y		4–8y		9–13y		14–16y		All subjects	
	Boys	Girls	Boys	Girls	Boys	Girls	Boys	Girls	Boys	Girls
*n*	278	264	735	710	697	679	412	369	2122	2022
BMI (kg/m^2^)	16.9 (1.5)	16.6 (1.5)	16.6 (2.0)	16.5 (2.0)	18.8 (3.2)	19.7 (4.0)	21.1 (3.2)	21.7 (3.5)	18.2 (3.1)	18.5 (3.7)
Urban (%)	72.9	73.9	69.6	68.9	68.2	66.9	70.5	69.9	69.7	69.0
Energy (kJ)	6289 (1246)	5913 (1177)	7808 (1556)	7026 (1469)	9696 (2150)	8350 (1815)	11574 (26442)	8910 (2032.2)	8960 (2610)	7669 (1944)
Total fat (g)	51.1 (14.8)	48.2 (13.5)	64.0 (18.8)	58.1 (18.4)	80.2 (24.2)	69.0 (20.3)	97.5 (28.1)	76.0 (23.6)	74.2 (26.8)	63.7 (21.5)
Saturated fat (g)	23.9 (8.3)	22.3 (7.4)	29.1 (9.9)	26.3 (9.4)	36.1 (12.3)	31.0 (10.4)	43.0 (14.3)	33.3 (12.4)	33.4 (13.1)	28.6 (10.8)
Total sugar (g)	100.9 (28.8)	92.1 (27.1)	117.8 (36.2)	105.2 (32.4)	138.6 (47.7)	121.9 (43.1)	155.1 (54.1)	124.6 (44.2)	129.7 (46.7)	112.6 (39.6)
Added sugar (g)	35.9 (21.9)	33.0 (20.5)	52.6 (27.3)	46.9 (24.8)	73.2 (36.1)	62.6 (33.2)	85.6 (44.9)	67.0 (35.7)	63.6 (37.5)	54.0 (31.7)
Fibre (g)	16.6 (5.9)	15.5 (5.7)	19.6 (6.4)	18.1 (5.5)	23.1 (8.0)	20.7 (7.4)	26.8 (9.3)	22.0 (7.7)	21.8 (8.2)	19.3 (6.9)

Values were presented as mean (SD) except for urban (%), which was presented as percentages. Data were weighted to account for over- or under-sampling to enable representation of the Australian population aged 2–16 years in terms of age group, gender and region.

### Impact on population intake of energy, sugar, total fat, saturated fat and fibre

Table 2 illustrates the overall change in intakes of energy, total sugar, added sugar, total fat, saturated fat and fibre resulting from the reformulation strategies. Decreases in energy, total sugar, added sugar and increases in total fat, saturated fat and fibre are observed in all groups. The greatest change is observed in 25% reformulation as expected with a projected mean reduction of 114 (92) kJ and 11.73 (7.52) g added sugar and an increase of 0.23 (0.32) g fat and 1.70 (1.78) g fibre daily.

**Table 2:** Overall change in daily dietary intake of energy and selected macronutrients, stratified by percentage reduction of added sugar.

	10% reduction		15% reduction		25% reduction	
	Mean (SD)	*p* value	Mean (SD)	*p* value	Mean (SD)	*p* value
Energy (kJ)	−45 (36)	<0.001	−68 (55)	<0.001	−114 (92)	<0.001
Total sugar (g)	−4.57 (2.94)	<0.001	−6.89 (4.43)	<0.001	−11.61 (7.47)	<0.001
Added sugar (g)	−4.62 (2.96)	<0.001	−6.97 (4.47)	<0.001	−11.73 (7.52)	<0.001
Total fat (g)	0.09 (0.12)	<0.001	0.13 (0.19)	<0.001	0.23 (0.32)	<0.001
Saturated fat (g)	0.5 (0.07)	<0.001	0.07 (0.11)	<0.001	0.12 (0.19)	<0.001
Fibre (g)	0.66 (0.69)	<0.001	1.00 (1.05)	<0.001	1.70 (1.78)	<0.001

Values were presented as mean (SD). Data were weighted to account for over- or under-sampling to enable representation of the Australian population aged 2–16 years. Negative value indicates decrease in intake.

*p* values for difference between intake under the reduction level *vs*. original intake, tested using paired sample *t*-test.

Table 3 shows the change across age groups in absolute amounts. The largest change was observed in 25% reformulation for 14–16 years old with a projected 161 (124) kJ and 15.3 (9.33) g added sugar reduction daily.

**Table 3:** Change in daily dietary intake of energy and selected macronutrients, stratified by percentage reduction of added sugar and age group.

	2–3y		4–8y		9–13y		14–16y	
	Mean (SD)	*p*	Mean (SD)	*p*	Mean (SD)	*p*	Mean (SD)	*p*
**10% reduction**								
Energy (kJ)	−23 (20)	*	−36 (25)	*	−53 (36)	*	−63 (49)	*
Total sugar (g)	−2.60 (1.73)	*	−3.87 (2.21)	*	−5.30 (2.90)	*	−5.97 (3.67)	*
Added sugar (g)	−2.64 (1.75)	*	−3.91 (2.24)	*	−5.36 (2.93)	*	−6.03 (3.69)	*
Total fat (g)	0.04 (0.07)	*	0.08 (0.12)	*	0.10 (0.13)	*	0.11 (0.13)	*
Saturated fat (g)	0.02 (0.04)	*	0.04 (0.07)	*	0.05 (0.08)	*	0.06 (0.8)	*
Fibre (g)	0.36 (0.41)	*	0.60 (0.62)	*	0.77 (0.75)	*	0.79 (0.78)	*
**15% reduction**								
Energy (kJ)	−35 (30)	*	−54 (37)	*	−80 (54)	*	−95 (74)	*
Total sugar (g)	−3.92 (2.61)	*	−5.83 (3.33)	*	−7.99 (4.38)	*	−9.01 (5.53)	*
Added sugar(g)	−3.97 (2.64)	*	−5.90 (3.37)	*	−8.07 (4.41)	*	−9.09 (5.55)	*
Total fat (g)	0.70 (0.11)	*	0.12 (0.19)	*	0.16 (0.20)	*	0.16 (0.20)	*
Saturatedfat (g)	0.04 (0.06)	*	0.07 (0.11)	*	0.08 (0.12)	*	0.09 (0.12)	*
Fibre (g)	0.55 (0.63)	*	0.91 (0.94)	*	1.16 (1.13)	*	1.19 (1.19)	*
**25% reduction**								
Energy (kJ)	−60 (51)	*	−90 (63)	*	−135 (91)	*	−161 (124)	*
Total sugar (g)	−6.60 (4.40)	*	−9.80 (5.61)	*	−13.45 (7.37)	*	−15.16 (9.30)	*
Added sugar (g)	−6.69 (4.46)	*	−9.93 (5.68)	*	−13.59 (7.43)	*	−15.30 (9.33)	*
Total fat (g)	0.11 (0.18)	*	0.21 (0.32)	*	0.27 (0.34)	*	0.28 (0.34)	*
Saturated fat (g)	0.06 (0.11)	*	0.11 (0.19)	*	0.14 (0.20)	*	0.15 (0.21)	*
Fibre (g)	0.93 (1.07)	*	1.54 (1.59)	*	1.97 (1.92)	*	2.02 (2.02)	*

Values were presented as mean (SD). Data were weighted to account for over- or under-sampling to enable representation of the Australian population aged 2–16 years. Negative value indicates decrease in intake. Sample size for each age group is as follow: 2–3 y (*n* = 542), 4–8 y (*n* = 1446), 9–13 y (*n* = 1376), 14–16 y (*n* = 781).

*indicates *p* < 0.001 for difference between intake under the reduction level of the age group *vs*. original intake of the same age group, tested using paired sample *t*-test.

Table 4 shows the change across age groups in percentage difference. A greater projected change in percentage intakes was observed in the older age group than the younger group and most of the differences were statistically significant. For example, total sugar intake under 25% reformulation decreased by 10.50% in 14–16 years old and 6.63% in 2–3 years old, while for added sugar intake, few differences were seen between age groups.

**Table 4:** Percentage change in daily dietary intake of energy and selected macronutrients, stratified by percentage reduction of added sugar and age group.

	10% reduction					15% reduction					25% reduction				
	2–3y	4–8y	9–13y	14–16y	Total	2–3y	4–8y	9–13y	14–16y	Total	2–3y	4–8y	9–13y	14–16y	Total
Energy (%)	−0.38^a^	−0.48^b^	−0.58^c^	−0.61^c^	−0.52	−0.57^a^	−0.72^b^	−0.87^c^	−0.92^c^	−0.79	−0.97^a^	−1.21^b^	−1.47^c^	−1.55^c^	−1.33
Total sugar (%)	−2.61^a^	−3.39^b^	−3.98^c^	−4.14^c^	−3.62	−3.94^a^	−5.10^b^	−6.00^c^	−6.24^c^	−5.46	−6.63^a^	−8.59^b^	−10.10^c^	−10.50^c^	−9.19
Added sugar (%)	−7.40^a^	−7.72^b^	−7.79^b^	−7.67^ab^	−7.69	−11.15^a^	−11.63^b^	−11.74^b^	−11.56^ab^	−11.59	−18.75^a^	−19.57^b^	−19.76^b^	−19.45^ab^	−19.50
Total fat (%)	0.09^a^	0.13^b^	0.14^b^	0.13^b^	0.13	0.14^a^	0.19^b^	0.21^b^	0.20^b^	0.19	0.23^a^	0.33^b^	0.36^b^	0.34^b^	0.33
Saturated fat (%)	0.10^a^	0.15^b^	0.16^b^	0.16^b^	0.15	0.15^a^	0.23^b^	0.24^b^	0.24^b^	0.23	0.26^a^	0.39^b^	0.41^b^	0.41^b^	0.38
Fibre (%)	2.65^a^	3.57^b^	3.93^b^	3.68^b^	3.59	4.02^a^	5.40^b^	5.96^b^	5.58^b^	5.44	6.84^a^	9.18^b^	10.13^b^	9.47^b^	9.25

Values were presented as mean. Data were weighted to account for over- or under-sampling to enable representation of the Australian population aged 2–16 years. Negative value indicates decrease in intake. Sample size for each age group is as follow: 2–3 y (*n* = 542), 4–8 y (*n* = 1446), 9–13 y (*n* = 1376), 14–16 y (*n* = 781).

*p* values for difference between intake under the reduction level of the age group *vs*. intake under same reduction level of other age group, tested using Bonferroni *post hoc* test following ANOVA. Values with different superscript are significantly different at *p* < 0.001.

Table 5 shows the projected changes in intake between men and women under the different percentage of reformulation in absolute and percentage difference respectively. A greater change in intake is observed in the higher percentage reformulation as expected in both men and women. Under 25% reformulation, 126 (101) kJ and 103 (81) kJ reduction per day are predicted in men and women, respectively, and a reduction in added sugar of 12.63 (8.07) g and 10.80 (6.77) g. No significant differences in percentage change in intake after reformulation between men and women.

**Table 5:** Change in daily dietary intake of energy and selected macronutrients, stratified by percentage reduction of added sugar and sex.

	10% reduction						15% reduction						25% reduction					
	Boys			Girls			Boys			Girls			Boys			Girls		
	Mean (SD)	%∆	*p*	Mean (SD)	%∆	*p*	Mean (SD)	%∆	*p*	Mean (SD)	%∆	*p*	Mean (SD)	%∆	*p*	Mean (SD)	%∆	*p*
Energy (kJ)	−49 (39)	−0.53	*	−40 (32)	−0.51	*	−75 (60)	−0.80	*	−61 (48)	−0.77	*	−126 (101)	−1.36	*	−103 (81)	−1.31	*
Total sugar (g)	−4.92 (3.16)	−3.65	*	−4.21 (2.65)	−3.60	*	−7.42 (4.76)	−5.50	*	−6.34 (3.99)	−5.43	*	−12.49 (8.02)	−9.26	*	−10.67 (6.72)	−9.13	*
Added sugar (g)	−4.98 (3.18)	−7.66	*	−4.26 (2.67)	−7.72	*	−7.50 (4.79)	−11.55	*	−6.41 (4.02)	−11.63	*	−12.63 (8.07)	−19.44	*	−10.80 (6.77)	−19.57	*
Total fat (g)	0.09 (0.13)	0.12	*	0.09 (0.12)	0.13	*	0.14 (0.20)	0.19	*	0.13 (0.18)	0.20	*	0.23 (0.33)	0.32	*	0.22 (0.31)	0.34	*
Saturated fat (g)	0.05 (0.08)	0.14	*	0.05 (0.07)	0.16	*	0.07 (0.11)	0.21	*	0.07 (0.11)	0.24	*	0.12 (0.20)	0.36	*	0.12 (0.19)	0.41	*
Fibre (g)	0.71 (0.75)	4.22	*	0.60 (0.61)	3.99	*	1.08 (1.14)	5.57	*	0.91 (0.93)	5.30	*	1.83 (1.94)	9.47	*	1.55 (1.58)	9.01	*

Values were presented as mean (SD). Data were weighted to account for over- or under-sampling to enable representation of the Australian population aged 2–16 years.

Negative value indicates decrease in intake. Sample size: boys (*n* = 2122), girls (*n* = 2022). No sex difference was observed.

*Indicates *p* < 0.001 for difference between intake under the reduction level of the sex *vs*. original intake of the same sex, tested using paired sample *t*-test.

## Discussion

The existing scientific consensus suggests that population diets could be improved if processed foods were reformulated to remove “empty calories” from added sugars and thus reduce energy density. The current study demonstrates that theoretically, this is possible through universal reformulation of the added sugar content of processed foods, however we believe the difficulties and effectiveness also need to be considered. Our modelling suggests that a decreased energy intake of ~114 kJ/day among Australian children could be achieved by reformulating specific processed foods through four strategies: reduction in added sugar without replacement/replacement with NNS alone (strategy 1), with NNS & polyols (strategy 2), with NNS & fibre (strategy 3) and with NNS & maltodextrin (strategy 4). The percentage reduction was greatest in older children as their consumption of added sugar was highest, and, thus more affected by the sugar reformulation.

In undertaking this modelling around reformulation, we have considered four possible reformulation strategies according to existing literature and industry practices. When the added sugar content of a food product is reduced, the loss in sweetness and/or functionality needs to be replaced to produce a product that the consumer will accept^8^. Replacement of sweetness could be done via the use of NNS, although concerns regarding their long-term safety as well as the undesirable aftertaste of some NNS has made widespread use of them as a sugar replacer an unattractive option for the food industry. Also, most NNS are unable to replace the functional properties of sugars^8^. The functional properties of sugars in food processing could be replaced by the use of bulking agents and humectants, such as polyols, fibres and maltodextrins. These could compensate for the functions of sugar that influence the sensory properies of foods, for example, tenderizing bakery products, affecting ice and crystallization in frozen products, mouthfeel in beverages, etc^8^. Each food group in our study was considered separately and assigned the most feasible strategy as mentioned in the method section. In real life, however, the reformulation of sugar in each food product will require different and specific methods and intensive testing which is beyond the scope of this study. The four strategies used are a simplified approach that we hope will provide a somewhat realistic estimation of the potential impact on population nutrients intake by sugar reformulation.

Overall, the resultant mean reduction in energy is small at the individual level (114 kJ/day), given the amount of reformulation required to achieve this total. However, one may argue that the additive effect of such small reductions in the longer term may still provide significant public health benefits at a population level^10^. A recent study published in 2016 modelled the possible impact in UK adults if sugar in SSBs is reduced by 15–30%. Their result suggested this could lead to reduction of 144 383 individuals with obesity and 19 094 incident cases of type 2 diabetes per year^11^. Since their results are based on the daily reduction in energy intake of 9–10 kcal (37.7–41.8 kJ) from SSBs only, the possible protective effect is expected to be greater if other foods are also reformulated as hypothesized in our study (energy reduction 42.93–109.12 kJ/day). Another modelling study also in UK adults suggested that a gradual reduction of added sugar in SSBs by 40% over 5 years could result in an average reduction of 38.4 kcal/day (161 kJ/day) and a significant decrease in the obesity prevalence and type 2 diabetes incidence^7^. Since the average consumption of free sugar by Australian and British populations are similar (about 60 g/day from survey carried out around 2011–12), the impact estimated in these UK analyses may also be applicable to Australia^12,13^. The impact may be even greater in children and adolescents. A study published by our group recently also found the total free sugar consumption by Australian children/adolescents (41–88 g/day) and adults (51–79 g/day) are comparable^4^, meaning that similar gains could be expected in Australian children. However the true health gain for children could be expected to be of even greater magnitude considering they will be subjected to the lower sugar intake for a longer period before those diseases are likely to emerge.

The population health benefits seem large under sugar reformulation, however, the efforts required in reformulating processed foods require careful consideration. As mentioned above, sugar provides a variety of functions in food manufacturing which require strategies more than merely replacing sugar with NNS to compensate for the sweetness. A considerable amount of resources may be needed to design formulation strategies and the resulting benefits may be hindered by a lower than 25% reduction achieved or lack of consumers’ acceptance due to the change of taste or texture of reformulated foods. Considering the limited resources, targeting foods that are easier to be reformulated, such as SSBs and candies, may be more cost effective. Some of these foods are also the highest contributors to the added sugars intake of the population^4^.

Diverse opinions were raised among food manufacturers on the recent Sugar Reduction Programme by Public Health England. Some argued that it is impossible to reduce 20% of sugar in the foods while others were in support of the program^14^. Cost effectiveness analysis should be carried out for evidence on whether food reformulation could provide a significant benefit in the population or whether resources may be better spent in other areas, such as portion control, developing better food labelling system, education, healthy diet promotion, or dealing with disparities in access to healthy foods. Food reformulation should not be the only focus on action on diet, obesity, and health.

Our study has several notable strengths. First, we considered various reformulation options – including different strategies based on the existing literature or industry practise, and different percentage reductions of added sugars amongst the strategies – this allowed a more credible estimation of the potential impact on energy and nutrient intake. Second, dietary intake data of a population based national survey were used to estimate the change in intake of Australian children which provided a realistic effect of reformulation on a representative population.

There are however limitations to our study. First, as it is a theoretical study, the current study did not test the sensory properties of food following reformulation, and as such we have made an assumption that the individuals will consume the same amount of the reformulated products. In reality, consumers may switch to another product, or change the amount they consume when the added sugars level of the product is altered. Taste, texture and price of food after reformulation will influence consumption at the population level. Consumers may also add table sugar to reformulated products to overcome the decreased sweetness. Future studies should, therefore, include consumer research and sensory analysis to address these issues and determine consumer acceptability. Second, there are many more reformulation strategies other than the four we considered and assigned to various food groups. Each food item may have different properties and require specific formula. The impact of reformulation may differ according to the formula chosen by the food manufacturers. Further investigation could also be done to estimate the potential improvements in health outcomes related to sugar intake following reformulation, such as obesity, diabetes and dental problems, which could provide valuable information on the effectiveness of reformulation on population health. The cost of reformulating different foods could also be investigated to provide cost effectiveness analysis.

To conclude, when added sugars were removed without replacement, or replaced with NNS with or without polyols, fibre or maltodextrin, the theoretical modelled energy and added sugar intake of Australian children and adolescents decreased. Although the magnitude of reduction is small at the individual level, the impact may be meaningful at the population level. Future works could focus on the resources needed to reformulate selected food groups and to estimate population health impact to assess the effectiveness of this approach. On top of that, food reformulation should not be the only focus for tackling the problem of high sugar intake while other approaches may provide more cost effective solutions, one among which could be habituating a lesser consumption thorugh behaviour modification.

## Methods

### Data source

Data from the 2007 Australian National Children’s Nutrition and Physical Activity Survey (2007ANCNPAS)^15^ were used to model the impact of the reformulations on usual dietary behaviour of Australian children. Details of the methodology and questionnaires used in the 2007ANCNPAS are available in the User’s Guide^15^. Children and adolescents aged 2–16 years were included in the survey, and categorised into the following age groups: 2–3 years, 4–8 years, 9–13 years, and 14–16 years. In total, 4 834 respondents were interviewed for the survey, and dietary intake data were assessed using two 24 hour recalls (one computer assisted personal interview, and one computer assisted telephone interview), collected 7–21 days apart (2 days data available for 4 608 respondents). Dietary intake were analysed using the AUSNUT2007 food composition database^16^.

### Data cleaning

For the current study, only respondents who completed two days of 24 hour recall were used. Extreme low and high reporters were identified using the Goldberg cut-off for specific physical activity level (PAL) criteria^17^. There was no information on PAL for children aged 9 years or below as they were too young to recall their physical activity level accurately, and a default PAL of 1.55 was used. Of the total respondents, 339 (7.0%) were considered extreme low reporters, and 129 (2.7%) were considered extreme high reporters^18^. The final dataset included 4 140 respondents, of which 49.6% were females.

### Dietary modelling

A systematic 10-step methodology was employed to estimate the added sugar content of foods in the AUSNUT2007 database^19^. After that, foods were categorised as ‘processed’ or ‘unprocessed’. Unprocessed foods such as fruits and vegetables were not considered to have added sugars^19^. For the purpose of the present study, pure sugars and honey were categorised as ‘unprocessed’ as reformulation of these foods is not possible (*n* = 6). Only processed foods (*n* = 890) with at least 5 g of added sugars per 100 grams were included in the modelling. Foods with <5 g added sugar/100 g were not modelled for reformulation in the current study as these would not contribute significantly to overall reductions.

Multiple percentage reductions were modelled to test the effects of reformulation at different levels. These levels were based on reformulated products quality, benefits or current effectiveness of reformulation, and challenges associated with reformulation^20^. Previous studies have investigated the effect of various levels of substitution on the aforementioned properties. The highest sugar reduction level with quality similar to that of the control products varies depending on the type of food and ingredients involved^21–25^. A level of 25% reduction in added sugar was specifically chosen as this may be of particular interest to food manufacturers. For example, in Australia and New Zealand, reduction of ≥25% of the original level is required to label a product as ‘reduced sugars’^26^. The Public Health England also proposed a 20% sugar reduction in a range of products by 2020^14^. A reduction above 25% was not performed in our models due to technical difficulties and the feasibility of such a high level reduction on food properties and consumer acceptance. For example, higher level of replacement of sugar could lead to lower cake quality in terms of the bubble formation, bulk density, crust colour, etc., which subsequently resulted in a lower acceptability in sensory analysis^25^.

All recorded foods were grouped on the basis of the sub-major food groups in the AUSNUT2007 database^19^. The four possible reformulation strategies were considered for each food group, and each group was assigned one of the strategies according to a balance of the most feasible functional replacement, consumer acceptance and the health benefits. Examples of the assignment are shown in Table 6 and the full list of assignments is shown in Supplementary Table 1.

**Table 6:** Examples of reformulation strategies assignment.

Strategy	Examples Food Groups	Reason
No substitution or with NNS/Sweetness enhancer only	Soft Drinks/Energy Drinks	The major use of sugar in these drinks is for sweetness which could be replaced by NNS.
	Breakfast Cereal, Hot Porridge Type	Sugar free hot porridge is common in the market where added sugar is mainly for the sweetness which could be replaced by NNS.
	Fruit juice with added sugar	The major use of sugar in juice is for the sweetness which could be replaced by NNS.
NNS + polyols	Frozen Milk Products (ice-cream, yogurt)	Polyols can depress freezing point and inhibit crystallization of other sugars which allow the frozen products to have similar scooping properties^34^.
	Baked goods (Breads, Biscuits, cakes)	Polyols can act as bulking agent and humectant which have a positive effect on texture and volume of baked goods such as biscuits and cakes, and yields products with similar sensory characteristics to the control products^28^.
NNS + 50% fibre	Chocolate	Inulin can act as a bulk ingredient in chocolate^29^.
	Tea, Coffee	Soluble fibre such as inulin could provide desire mouthfeel in certain drinks mimicking sugar. The fibre used is usually odourless, and has a bland flavour which won’t affect the taste^9^.
	Salad Dressings	Inulin is used in low fat salad dressing replacing fat to provide body and mouthfeel^35^. We assume it could also be used to replace the texture loss which was contributed by sugar.
	Breakfast cereals	Inulin and oligofructose could provide crispness and expansion to cereals which are desired characteristics^29^.
NNS + 50% maltodextrin	Cream/Custards	Maltodextrin could provide a creamy mouthfeel^36^.

The four strategies used were:

*Strategy 1 – added sugars reduced, and not replaced by other macronutrients/replaced by NNS only (n* = **197**): This strategy was chosen to demonstrate the effects of simple removal of added sugars on the nutrient profile of foods. It was postulated that by reducing added sugars, all nutrients will subsequently concentrate and therefore increase on a per 100 g basis. Of note is the fact that the final added sugars level per 100 g of the reformulated product would be higher than expected due to the concentration effect. The replacement with NNS is used for adding sweetness and is assumed to have no effect on the final weight. As an example, for a product with 50 g added sugars per 100 g, it is expected a 10% reduction would result in a product with 50 × 90% = 45 g added sugars per 100 g. However, in reality, due to the concentration effect, the final added sugars content will be $\frac{(\mathrm{50}\times \mathrm{90} \% )\,\times \mathrm{100}}{\mathrm{100}-\,(\mathrm{50}\times \mathrm{10} \% )}={\rm{47}}{\rm{.4}}\,{\rm{g}}\,{\rm{per}}\,{\rm{100}}\,{\rm{g}}$.

*Strategy 2 – replacement with polyols and NNS (n* = **393)**:There is a range of polyols including erythritol, lactitol and xylitol. They can replace the sugar, for its bulking, humectant and thickening properties which are crucial in food manufacturing and consumers’ acceptance^27^. They also have lower calories (10 kJ/g vs 17 kJ/g) and glycaemic indices than sugar, while possessing prebiotic and anti-caries functions. Polyols have already been used as additives in certain food products such as hard candies and chewing gums^27^. Their sweetness is lower than sucrose hence NNS can be used to replace the loss in sweetness. It has been shown that polyols plus NNS could be used to replace sugar in a 1:1 ratio while giving acceptable results in sensory evaluations^28^.

*Strategy 3 – replacement with fibre and NNS (n* = **267)**:Dietary fibre is another potential sugar replacement, with inulin being most commonly used as it can provide mouthfeel, texture, moisture retention and heat resistance^29^. From previous studies, a reasonable sensory score could be obtained by replacing 50% of sugar removed with inulin and NNS in certain foods, e.g. chocolate and muffins^30,31^. Therefore, we assumed a 50% replacement is feasible, e.g. adding 5 g of inulin for 10 g of sugar removed.

*Strategy 4 – replacement with maltodextrin and NNS (n* = **33)** Maltodextrin is used as a bulking agent by the food industry. It has a bland flavour and low sweetness. Relatively lower price is the major advantage over other bulking agents^9^. However, maltodextrin can be fully digested and has similar energy as glucose and thus provides no extra benefits in terms of energy. Only a small number of food groups were assigned this strategy. Studies have shown that replacement of 25% – 75% sugar by maltodextrin (and NNS for sweetness) can produce products with similar sensory scores as normal products, for example in milk chocolate^30^. Therefore, we assumed the medium of the range, i.e. 50% replacement is suitable, e.g. adding 5 g of maltodextrin for 10 g of sugar removed.

The formulae used for calculating changes in the nutritional composition of individual foods for each strategy are presented in Supplementary File 1.

Next, the revised nutritional compositions of the ‘reformulated’ foods were used to model the likely impact of these reformulations on the diets of Australian children and adolescents particularly on intakes of energy, sugar, total fat, saturated fat and fibre. In the modelling, an assumption was made that consumers will consume the same amount by weight of the reformulated product, which mimics the theoretical effect of ‘stealth reduction’ whereby a negative nutrient (e.g. salt) is reduced without the consumers noticing the change^32^.

### Statistical analysis

Data were weighted to ensure results were representative of the Australian children and adolescents population. Paired sample *t*-tests were used to examine the change in intakes of energy, total sugar, added sugar, total fat, saturated fat and fibre resulting from the reformulation compared with the original formulation. Results after stratification by age group and sex were also presented. As the absolute change in intake across age groups and sex may be due to different amounts of baseline intake, percentage changes were also calculated and compared using ANOVA (for age group) and independent sample *t*-test (for sex). Bonferroni *post hoc* analysis was conducted after ANOVA to test for differences between any two age groups. Levene’s test was carried out to check for difference in variance in intakes between men and women before independent sample *t*-test for sex. Due to the number of comparisons made, *p* < 0.001 was considered statistically significant to minimize type I error in *t*-test and ANOVA while *p* < 0.05 was considered significant for Levene’s test^33^. All statistical analyses were performed using Statistical Packages for Social Science version 22.0 (SPSS Australasia Pty Ltd, North Sydney, NSW Australia).

## Electronic supplementary material

Supplementary Information
