# Supplementary material for: Modelling of the impact of universal added sugar reduction through food reformulation
Source: Sci Rep. 2017 Dec 12;7:17392. doi: 10.1038/s41598-017-17417-8 (PMC5727294; doi:10.1038/s41598-017-17417-8)
Supplement: Supplementary file 1 — Supplementary Information [file 41598_2017_17417_MOESM1_ESM.pdf]

Supplementary information

Title: Modelling of the impact of universal added sugar reduction through food reformulation

Authors: Chris Ho Ching Yeung, Paayal Gohil, Anna M Rangan, Victoria M Flood, Jayashree Arcot, Timothy P Gill, Jimmy Chun Yu Louie

Supplemental Information – Equations used for the 4 reformulation strategies

Strategy 1: No substitution or with NNS/Sweetness enhancer only

$$\text{New fat per 100 g} = \frac{\text{original fat per 100 g} \times 100}{100 - (\text{original added sugars per 100 g} \times \%\ \text{reduction})} \text{ -- (1)}$$

$$\text{New saturated fat per 100 g} = \frac{\text{original saturated fat per 100 g} \times 100}{100 - (\text{original added sugars per 100 g} \times \%\ \text{reduction})} \text{ ---------------------------- (2)}$$

$$\text{New protein per 100 g} = \frac{\text{original protein per 100 g} \times 100}{100 - (\text{original added sugars per 100 g} \times \%\ \text{reduction})} \text{ ---------------------------------- (3)}$$

$$\text{New total sugar per 100 g} = \frac{[\text{old total sugars per 100 g} - (\text{original added sugar per 100 g} \times \%\ \text{reduction})] \times 100}{100 - (\text{original added sugars per 100 g} \times \%\ \text{reduction})} \text{ ----- (4)}$$

$$\text{New added sugar per 100 g} = \frac{[\text{old added sugars per 100 g} \times (100\% - \%\ \text{reduction})] \times 100}{100 - (\text{original added sugars per 100 g} \times \%\ \text{reduction})} \text{ ----------------------- (5)}$$

$$\text{New fibre per 100 g} = \frac{\text{original fibre per 100 g} \times 100}{100 - (\text{original added sugars per 100 g} \times \%\ \text{reduction})} \text{ ------------------------------------- (6)}$$

Strategy 2: NNS + polyols

New fat per 100 g = *original fat per 100 g* -- (7)

New saturated fat per 100 g = *original saturated fat per 100 g* ----------------------------------- (8)

New protein per 100 g = *original protein per 100 g* -- (9)

New total sugar per 100 g = *original total sugar per 100 g – (original added sugars per 100 g × % reduction)* --- (10)

New added sugar per 100 g = *original added sugars per 100 g × (100% − % reduction)* --- (11)

New fibre per 100 g = *original fibre per 100* -- (12)

Polyols per 100g = *original added sugar per 100 g × (% reduction)* --------------------------- (13)

Strategy 3: NNS + 50% fibre

$$\text{New fat per 100 g} = \frac{\text{original fat per 100 g x 100}}{100 - (\text{original added sugars per 100 g} \times \%\ \text{reduction} \times 50\%)} \text{ --------------------------------- (14)}$$

$$\text{New saturated fat per 100 g} = \frac{\text{original saturated fat per 100 g x 100}}{100 - (\text{original added sugars per 100 g} \times \%\ \text{reduction} \times 50\%)} \text{ --------------------- (15)}$$

$$\text{New protein per 100 g} = \frac{\text{original protein per 100 g x 100}}{100 - (\text{original added sugars per 100 g} \times \%\ \text{reduction} \times 50\%)} \text{ --------------------------- (16)}$$

$$\text{New total sugar per 100 g} = \frac{[\text{original total sugars per 100 g} - (\text{original added sugars per 100 g} \times \%\ \text{reduction})] \times 100}{100 - (\text{original added sugars per 100 g} \times \%\ \text{reduction} \times 50\%)} \text{ (17)}$$

$$\text{New added sugar per 100 g} = \frac{[\text{original added sugars per 100 g x } (100\% - \%\ \text{reduction})] \times 100}{100 - (\text{original added sugars per 100 g} \times \%\ \text{reduction} \times 50\%)} \text{ -------------------(18)}$$

$$\text{New fibre per 100 g} = \frac{[\text{original fibre per 100 g} + (\text{original added sugars per 100 g} \times \text{\% reduction} \times 50\%)] \times 100}{100 - (\text{original added sugars per 100 g} \times \text{\% reduction} \times 50\%)} \text{ -------} \quad (19)$$

Strategy 4: NNS + 50% maltodextrin

$$\text{New fat per 100 g} = \frac{\text{original fat per 100 g} \times 100}{100 - (\text{original added sugars per 100 g} \times \text{\% reduction} \times 50\%)} \text{ --------------------------------} \quad (20)$$

$$\text{New saturated fat per 100 g} = \frac{\text{original saturated fat per 100 g x 100}}{100 - (\text{original added sugars per 100 g} \times \text{\% reduction} \times 50\%)} \text{ ----------------------} \quad (21)$$

$$\text{New protein per 100 g} = \frac{\text{original protein per 100 g} \times 100}{100 - (\text{original added sugars per 100 g} \times \text{\% reduction} \times 50\%)} \text{ ----------------------------} \quad (22)$$

$$\text{New total sugar per 100 g} = \frac{[\text{original total sugars per 100 g} - (\text{original added sugars per 100 g} \times \text{\% reduction})] \times 100}{100 - (\text{original added sugars per 100 g} \times \text{\% reduction} \times 50\%)} \quad (23)$$

$$\text{New added sugar per 100 g} = \frac{[\text{original added sugars per 100 g} \times (100\% - \text{\% reduction})] \times 100}{100 - (\text{original added sugars per 100 g} \times \text{\% reduction} \times 50\%)} \text{ -------------------} \quad (24)$$

$$\text{New fibre per 100 g} = \frac{\text{original fibre per 100 g} \times 100}{100 - (\text{original added sugars per 100 g} \times \text{\% reduction} \times 50\%)} \text{ ------------------------------} \quad (25)$$

Supplementary Table 1 - Reformulation strategies assignment

Strategy		Food groups
No substitution or with NNS/ Sweetness enhancer only	113	Fruit and vegetable juices, and drinks
	114	Cordials
	115	Soft drinks, and flavoured mineral waters
	116	Electrolyte, energy and fortified drinks
	126	Breakfast cereal, hot porridge type
	155	Fish and seafood products (homemade and takeaway)
	166	Other fruit
	167	Mixtures of two or more groups of fruits
	168	Dried Fruit, Preserved Fruit
	169	Mixed dishes where fruit is the major component
	186	Processed Meat
	187	Mixed dishes where beef, veal or lamb is the major component
	188	Mixed dishes where pork, bacon, ham is the major component
	189	Mixed dishes where poultry or game is the major component
	212	Dry soup mix
	213	Canned condensed soup (unprepared)
	222	Nuts and nut products
	232	Pickles, chutneys and relishes
	249	Dishes where vegetable is the major component
	252	Mature legume and pulse products and dishes
	262	Corn Snacks
	272	Jam and lemon spreads, chocolate spreads, sauces
	273	Dishes and products other than confectionery where sugar is the major component
	295	Pre-mixed drinks
NNS + polyols	122	Regular breads, and bread rolls (plain/unfilled/untopped varieties)
	123	English-style muffins, flat breads, and savoury and sweet breads
	131	Sweet biscuits
	132	Savoury biscuits
	133	Cakes, buns, muffins, scones, cake-type desserts
	134	Pastries
	135	Mixed dishes where cereal is the major ingredient
	136	Batter-based products
	172	Dishes Where Egg Is The Major Ingredient
	192	Yoghurt
	195	Frozen milk products
	197	Other dishes where milk or a milk product is the major compomemt
	204	Soy-based ice confection
	205	Soy-based yoghurts
	283	Other confectionery
NNS + 50% fibre	112	Coffee and coffee substitutes
	118	Other beverage flavourings and prepared beverages
	125	Breakfast cereals and bars, unfortified and fortified varieties
	198	Flavoured milks
	201	Dairy milk substitutes, unflavoured
	202	Dairy milk substitutes, flavoured
	233	Salad dressings
	281	Chocolate and chocolate-based confectionery
	282	Cereal-, fruit-, nut- and seed-bars
NNS + 50% maltodextrin	193	Cream
	196	Custards
	231	Gravies and savoury sauces
